# A case of dementia with Lewy bodies with psychosis induced by low-dose gabapentinoids

**DOI:** 10.1186/s12888-025-06937-7

**Published:** 2025-05-15

**Authors:** Hideki Kanemoto, Taisuke Akiyama, Daiki Taomoto, Manabu Ikeda

**Affiliations:** 1 Health and Counseling Center, The University of Osaka, 1-17 Machikaneyama-cho, Toyonaka, Osaka 560-0043 Japan; 2 Department of Psychiatry, The University of Osaka Graduate School of Medicine, Suita, Osaka Japan; 3 Minoh Neuropsychiatric Sanatorium, Minoh, Osaka Japan

**Keywords:** Dementia with lewy bodies, Pregabalin, Mirogabalin, Drug-induced psychosis, Visual hallucinations, Delusional misidentification, Irritability

## Abstract

**Background:**

Hypersensitivity to antipsychotic drugs is one of the supportive features of dementia with Lewy bodies, and side effects to drugs other than antipsychotics are also known to occur frequently. We experienced a case of dementia with Lewy bodies in which hallucinations and delusions repeatedly appeared and disappeared after administration and discontinuation of mirogabalin and pregabalin.

**Case presentation:**

The patient, a woman in her late 70s, developed hallucinations and delusional misidentification of places and persons immediately after receiving a prescription of mirogabalin (15 mg daily) for neuropathic pain. After discontinuation of mirogabalin, her hallucinatory delusions improved but remained. Mild dementia and mild parkinsonism were associated, cognitive fluctuations were evident, and dopamine-transporter scintigraphy showed bilateral striatal uptake reduction. Residual psychosis resolved with donepezil. Later, when the pain worsened, pregabalin (25 mg daily) was administered, and the psychosis recurred and resolved with discontinuation.

**Conclusions:**

Although pregabalin-induced psychosis has been reported at higher doses (300–450 mg daily), it has not been reported at doses as low as those used in this patient. Gabapentinoids may cause psychosis in patients with dementia with Lewy bodies even at low doses, likely due to hypersensitivity to gabapentinoids in DLB.

## Background

Dementia with Lewy bodies (DLB) is the second most common form of dementia after Alzheimer’s disease and is characterized by various symptoms, including visual hallucinations, cognitive fluctuations, parkinsonism, and rapid eye movement sleep behavioral disorder, from the early stages of the disease [1]. These symptoms can affect the patient’s daily life [2] and increase family care burden [3], making appropriate therapeutic interventions important. Interventions for psychosis and parkinsonism are prime examples.

Pharmacological treatment in patients with DLB is challenging owing to their hypersensitivity to drugs. Hypersensitivity to antipsychotics is cited as a supportive clinical feature of DLB [1], and even small doses can cause acute onset or exacerbation of parkinsonism and impaired consciousness [4]. Moreover, patients with DLB can show hypersensitivity to drugs other than antipsychotics. Anti-parkinsonian drugs [5] and zolpidem [6] have been reported to induce psychosis even in small doses, and administration of anticholinergics has been associated with a high risk of delirium [7]. Additionally, studies also suggest that older patients with major depression who subsequently convert to DLB are more likely to experience adverse effects with psychotropic medications, including antidepressants [8]. Therefore, the risk of side effects from a variety of drugs should be considered in patients with DLB.

Here, we report a case of DLB with psychosis that appeared after the administration of mirogabalin and pregabalin, which are medications used to treat neuropathic pain.

## Case presentation

The patient was a woman in her late 70s. She had been living with her daughter and son since her husband’s death > 10 years prior. Other than leg pain associated with scoliosis, for which she was prescribed mirogabalin (described below), she had no history of any other notable medical conditions, including psychiatric disorders.

Her son had also passed away a year before her initial visit to our hospital. Although her activities of daily living were independent, she had begun to show signs of forgetfulness in daily life. Three months earlier, she had visited an orthopedic surgeon for pain in her lower extremities, was diagnosed with scoliosis, and was prescribed mirogabalin (15 mg/day). Thereafter, she had complained frequently of visual hallucinations of her deceased son and husband, misidentified her daughter as her sister, and was irritable. She could not identify her home as the place where she lived, complained that she wanted to go home, and even visited her birthplace > 300 km away, alone. Suspecting dementia, her family took her to her general practitioner, who prescribed donepezil, Yokukansan (a Japanese herbal medicine), aripiprazole and olanzapine, which were ineffective. Three months after disease onset, she was referred to our clinic for further evaluation and treatment.

**Fig. 1:**
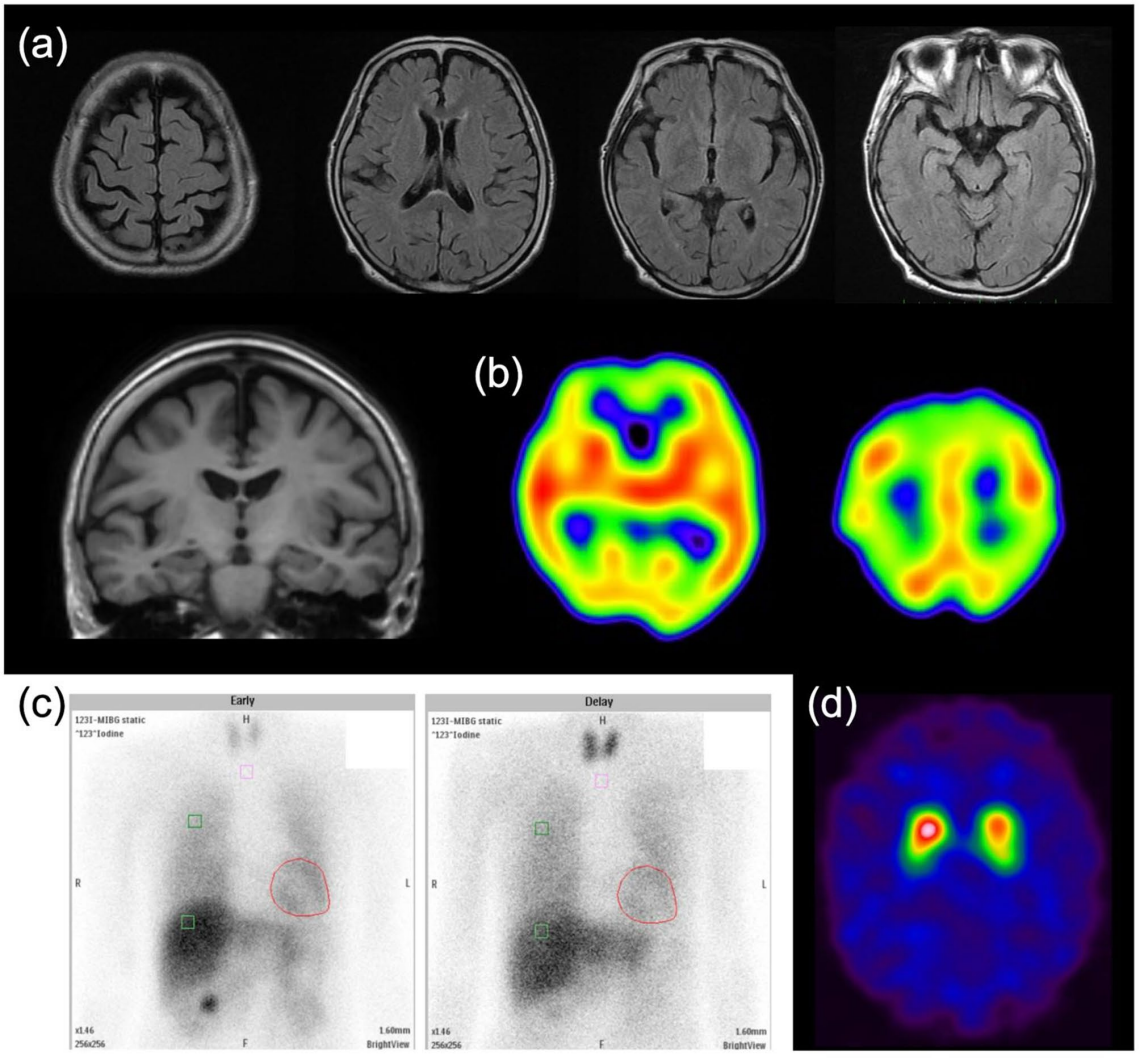
Neuroimaging findings in this patient with dementia with Lewy bodies. (**a**) Cranial magnetic resonance imaging showed no abnormalities. (**b**) N-isopropyl-p-iodoamphetamine single-photon emission computed tomography showed reduced perfusion in the bilateral parietal, frontal, precuneus, and occipital lobes. (**c**) ^123^I-meta-iodobenzylguanidine myocardial scintigraphy showed that the heart-to-mediastinum ratio was maintained at 3.69 and 2.41 in the early and late stages, respectively, but the washout rate was high at 59.5%, suggesting mild abnormality of cardiac sympathetic nerve function. (**d**) ^123^I-N-omega-fluoropropyl-2-betacarbomethoxy-3-beta (4-iodophenyl) nortropane dopamine-transporter single-photon emission computed tomography showed decreased uptake in the posterior striatum, especially on the left side

Her Mini-Mental State Examination (MMSE) score was 22, with suspected mild impairments in orientation, memory, attention, and visuospatial cognition. According to the family, her cognition fluctuated daily, with a score of 6 on the Mayo Fluctuations Scale [9]. Mild rigidity in the left upper limb and bradykinesia were noted; however, no other indications of parkinsonism were observed. Yokukansan (7.5 g), aripiprazole (2 mg), and mirogabalin (15 mg) were administered daily. Blood tests revealed nothing that could cause cognitive impairment or psychiatric symptoms. The symptoms suggested DLB; however, the patient’s history indicated that these cognitive and psychiatric symptoms were drug-induced. After the discontinuation of mirogabalin, the return-home behavior associated with the misidentification of places and her irritability disappeared. The visual hallucinations and misidentification of people reduced in frequency but persisted. In addition, no pain flare-ups were observed after discontinuing mirogabalin, probably owing to treatment with this drug over a period of 3 months. Magnetic resonance imaging of the brain was unremarkable. However, N-isopropyl-p-iodoamphetamine single-photon emission computed tomography showed reduced perfusion in the bilateral parietal, frontal, precuneus, and occipital lobes (Fig. 1a, b). ^123^I-meta-iodobenzylguanidine myocardial scintigraphy revealed that the heart-to-mediastinum ratio was maintained at 3.69 and 2.41 in the early and late stages, respectively, but the washout rate was high at 59.5%, suggesting mild abnormality of cardiac sympathetic nerve function (Fig. 1c). ^123^I-N-omega-fluoropropyl-2-betacarbomethoxy-3-beta (4-iodophenyl) nortropane dopamine-transporter single-photon emission computed tomography showed decreased uptake in the posterior striatum, especially on the left side (Fig. 1d).

Electroencephalography showed that the basic rhythm was 9–10 Hz occipital predominant, mixed with a generalized 6–7 Hz slow wave. However, no obvious epileptogenic findings were noted. Based on these findings, a diagnosis of probable DLB was made [1], and treatment with donepezil (3 mg) was initiated. When the dose was increased to 5 mg, the patient’s MMSE score increased to 25 points, and the misidentification of people disappeared. Aripiprazole was discontinued soon thereafter, but symptoms did not relapse. Residual insomnia improved with a prescription of quetiapine (12.5 mg).

Thereafter, cognitive impairment progressed slowly, and after 1 year, the MMSE score dropped to 20 points. Two years later, her back pain intensified, and she was prescribed 1,200 mg of acetaminophen and 100 mg of tramadol. However, the pain did not improve, and tramadol was discontinued. After being administered 25 mg of pregabalin, she began to exhibit strange behaviors: she became irritable, talked to herself, and prepared meals for four people, despite living with only her daughter. These behaviors disappeared after discontinuation of the pregabalin. Her day care service advised increased exercise therapy, and in subsequent outpatient visits, her complaints of pain had decreased.

The scores of the Adverse Drug Reaction Probability Scale [10] were 3 and 5 for mirogabalin and pregabalin, respectively (Table 1).

**Table 1:** The results of the adverse drug reaction probability scale

Question	Scores for	
	Mirogabalin	Pregabalin
1. Are there previous conclusive reports on this reaction?	0	1
2. Did the adverse event appear after the suspected drug was administered?	2	2
3. Did the adverse reaction improve when the drug was discontinued or a specific antagonist was administered?	1	1
4. Did the adverse event reappear when the drug was re-administered?	0	0
5. Are there alternative causes (other than the drug) that could on their own have caused the reaction?	0	0
6. Did the reaction reappear when a placebo was given?	0	0
7. Was the drug detected in blood (or other fluids) in concentrations known to be toxic?	0	0
8. Was the reaction more severe when the dose was increased or less severe when the dose was decreased?	0	0
9. Did the patient have a similar reaction to the same or similar drugs in any previous exposure?	0	1
10. Was the adverse event confirmed by any objective evidence?	0	0
Total score	3	5

## Discussion and conclusions

Here, we report a case of a patient who experienced delusions, hallucinations, and irritability that appeared and disappeared following the initiation and discontinuation of mirogabalin and pregabalin, which have similar mechanisms of action in the treatment of chronic pain. According to the Adverse Drug Reaction Probability Scale [10], the likelihood of drug-induced side effects was rated as “possible” for mirogabalin (score: 3) and “probable” for pregabalin (score: 5). The following reasons suggest that, despite the low scores, these drugs affected the patient’s psychiatric symptoms: (1) Some items (4, 6, and 7) on this scale are difficult to implement in general practice from an ethical viewpoint. Moreover, these tests are not yet routinely used in general practice. (2) In this case, the reactions appeared following the initial dose of pregabalin. Therefore, evaluation of symptom changes with dose increase or decrease, as required by the eighth item, was not possible. (3) Although the sixth item could not be evaluated because the response to placebo was not assessed, no similar response was observed when acetaminophen or tramadol was added for pain management. Therefore, in general clinical practice, low scores on such rating scales may still reflect actual adverse drug reactions and should not be used as a sole reason to rule out these adverse drug reactions.

Even without these medications, the patient met the clinical diagnostic criteria for DLB [1]: She showed mild generalized cognitive impairment, including two core features of cognitive fluctuation and mild parkinsonism, as well as abnormalities in the indicative biomarker dopamine transporter imaging. In DLB, visual hallucinations are seen early on as a core clinical feature, and delusions of misidentification are supportive clinical features [1]. Therefore, the psychosis observed in this case was characteristic of DLB. Notably, although her psychosis worsened and improved with initiation and discontinuation of medications, some symptoms persisted even after discontinuation of the medications and resolved only with donepezil, which is known to improve psychosis in DLB [11]. This suggests that her psychosis was influenced not only by gabapentinoids but also by DLB itself. Such reactions to drugs in DLB are similar to hypersensitivity to antipsychotics, which is a supportive feature of DLB. Hypersensitivity to antipsychotics in DLB is characterized by an acute onset or exacerbation of parkinsonism [4], a core clinical feature of DLB and a major side effect of antipsychotics. The reaction in this case could be described as hypersensitivity to mirogabalin and pregabalin in DLB. Additionally, DLB induces hypersensitivity reactions to other drugs. Drugs that produce delusions and hallucinations as side effects in DLB include anti-parkinsonian drugs [5] and zolpidem [6]. Owing to the effects of cholinergic neuron damage, the risk of delirium with anticholinergic drugs is also thought to be higher in patients with DLB [7].

Although pregabalin and mirogabalin are generally considered well-tolerated, safe drugs, their reported side effects include somnolence, dizziness, and headaches. Few reports have identified delusions, hallucinations, or other psychiatric symptoms as typical side effects of pregabalin, even in patients receiving low doses [12]. Pregabalin and mirogabalin bind to the α2-δ auxiliary subunit of presynaptic voltage-dependent calcium channels in the central nervous system [13, 14]. Strong binding to this site attenuates depolarization-induced calcium influx at nerve endings and reduces the release of excitatory neurotransmitters such as glutamate, noradrenaline, and substance P. In the rat model of neuropathic pain, chronic pain has been shown to decrease central dopamine function and induce depression-like behavior. However, repeated administration of gabapentin has been reported to modulate dopaminergic system activity and improve depression-like behavior [15]. In light of this, it is conceivable that pregabalin and mirogabalin may modulate the dopaminergic system activity. Although changes in these neurotransmitters may cause psychiatric symptoms such as hallucinations and delusions, the specific reason this patient developed psychotic symptoms while taking pregabalin and mirogabalin remains unclear.

Pregabalin-induced psychosis has been reported at higher doses (300–450 mg daily) [16, 17] but not at doses as low as those used for the current patient. This finding may indicate the hypersensitivity of DLB to pregabalin and mirogabalin. These drugs inhibit the release of neurotransmitters by blocking the influx of calcium ions into the presynaptic terminals, thereby producing analgesia [13, 18]. In DLB, the number of Lewy bodies in the cortex is low relative to the total number of neurons and does not correlate with the degree of cognitive impairment, whereas the number of alpha-synuclein aggregates is high in presynaptic terminals [19, 20]. Thus, patients with DLB may exhibit hypersensitivity to even small doses of pregabalin and mirogabalin, because the presynaptic terminals with alpha-synuclein aggregates are the acting sites of pregabalin and mirogabalin.

In a study of patients taking oxycodone for cancer pain, multivariate analysis revealed that the use of pregabalin or mirogabalin was a risk factor for opioid-induced neurotoxicity, including delirium and hallucinations [21]. The use of benzodiazepines or muscarinic antagonists was not a significant risk factor in this study. However, the above findings suggest that pregabalin, mirogabalin, benzodiazepines, and anticholinergics should be considered to have a high risk of inducing delusions and hallucinations in patients with DLB.

The temporary exacerbation of cognitive impairment and psychiatric symptoms seen in the current patient was drug-related and may resemble delirium in mechanism. Therefore, although she had presented with mild cognitive impairment that did not affect her daily life for approximately 6 months prior to the current episode, her condition was considered similar to delirium-onset prodromal DLB [22]. Of the five consecutive patients with delirium-onset DLB we described previously, a few were considered to be temporally close to the onset of delirium and cognitive impairment [23]. Thus, in patients with drug-induced psychiatric symptoms, the possibility of conversion to dementia should be considered.

In conclusion, this case report suggests that even low doses of pregabalin and mirogabalin may cause psychiatric symptoms, including psychosis and cognitive impairment, in patients with DLB. Furthermore, although psychosis triggered by such drugs may improve after drug discontinuation, some symptoms may remain and trigger the persistent onset of DLB symptoms. As these drugs are widely used for chronic pain, which is a frequent complaint in older people, recognizing that such reactions can occur even at low doses and that Lewy body disease may be present in the background are clinically important.

## Data Availability

No datasets were generated or analysed during the current study.

## Abbreviations

DLB: Dementia with Lewy bodies
MMSE: Mini-Mental State Examination
